# Observations on a Peculiar Kind of Traumatic Delirium

**Published:** 1830-04

**Authors:** M. Helis


### TRAUMATIC DELIRIUM.

### *Observations on a peculiar kind of Traumatic Delirium.* By M. Helis.

The duty of a surgeon consists not only in carefully preparing his patients for operation, or in operating with skill and courage, but he must also exert himself to lead them safely through the various dangers which arise after the performance of the operation. The moral and physical excitement experienced by a patient who has just been operated upon, exposes him to various maladies. But there is one kind of affection which usually occurs after surgical operations, and which appears to be particularly connected with them; and it is indispensable for the operator to be as well acquainted with such cases, in order that he may complete his task, as that he should be well skilled in anatomy to perform the operation. It is, indeed, by this kind of knowledge that the scientific surgeon distinguishes himself from those who merely possess a little manual dexterity, and who are ready to exclaim with a lithotomist of the last century, "I have operated, let Providence complete the cure." From the slightest wounds the most formidable symptoms not unfrequently arise. It behoves us, then, to watch a patient with the greatest attention who has been the subject of an operation in which the most important parts have been divided. After wounds, whether inflicted by accident or the knife of the surgeon, the nervous system is often so much disturbed as to lead to a species of delirium, the precise causes of which are obscure. This mental disturbance varies in its progress: the symptoms which accompany it are sometimes very alarming, but nervous or traumatic delirium is seldom fatal, if properly treated.

The object M. Helis has in view is to illustrate by cases the advantage of the treatment recommended by M. Dupuytren, which we have ourselves seen employed with the best effect.

Case I. *Delirium after the Operation for Sarcocele.*

M. D. R., twenty-five years of age, of a nervous temperament, was operated upon for sarcocele of a very large size in 1817. The day after the operation, he was restless, and very much alarmed lest hemorrhage should occur. The succeeding day his agitation had much increased; the slightest word or movement produced the greatest degree of excitement; the least sensation redoubled his apprehensions. Still his progress was satisfactory. He soon complained, however, of pains in his limbs and chest; his eyes glistened, he breathed quickly, eagerly demanded food, and was determined to rise from bed. His mind wandered, he repulsed those who were the most attentive to him, and called loudly for his family: his whole body was incessantly in motion. His cries, the appearance of his eyes, the fixed state of the pupil, his face covered with sweat, and his calm and regular pulse during all his agitation, convinced M. Dupuytren of the nature of the case. Particular attention, however, was paid to the state of the chest, as the patient complained of a fixed pain in that part.

When it was ascertained that he was labouring under no pulmonic disease, an enema with six drops of laudanum was immediately given; and the patient was ordered to be kept perfectly quiet, and undisturbed by friends. In an hour after the administration of the enema, M. D. R. ceased to ramble, and fell into a sound sleep, which lasted for several hours. The cure was complete in twenty-five days.

Case II. *Delirium after Luxation of the Femur.*

A mason fell from a scaffold, and luxated the left femur. He was carried immediately to the Hôtel Dieu, and the next day the luxation was reduced with the utmost facility. The patient was dreadfully alarmed at the apparatus which was employed, and could not believe himself so speedily cured. On the following day he was extremely agitated. The eyes were unnaturally brilliant, and turgid with blood; his face red, and covered with sweat. He cried out incessantly, and endeavoured to break the bandages by which his limb was secured. In the midst of this mental derangement, the pulse was full, regular, and natural in number; temperature of the skin not increased.

The sister of the ward, who was well accustomed to these symptoms, ordered an enema with ten drops of laudanum in it to be immediately given; and no other remedy was necessary to restore the patient to reason.

Case III. *Delirium after the Fracture of a Rib.*

Langlois, a mason, was admitted into the Hôtel Dieu with fracture of a rib. A tight bandage was placed round his body. From the facility with which such accidents are usually cured, but little attention was paid to this patient, but on the third day he was attacked with delirium, which continued unabated. He threw himself into various positions: all his muscles were in a state of continued tension:

eyes very brilliant; skin covered with sweat; pulse natural. He fancied he saw images dancing before him in the air, and that various experiments were being tried upon his bed.

As this man was of a full habit of body, he was first bled, but without relief. An enema with ten drops of laudanum was then given, and a slight degree of calmness succeeded. The next day this dose was doubled, without advantage. His cries and constant restlessness were now so annoying to the other patients, that he was put in a ward by himself, and forty drops of laudanum were given in an enema, and the delirium speedily subsided.

Case IV. This patient had attempted to commit suicide by cutting his throat. On the second day after the attempt he became delirious, and it was necessary to restrain him by the strait-waistcoat.

An anodyne was first given by the mouth, with but little effect; but an enema with a few drops of laudanum quickly restored him to reason. On the fourth day the wound assumed a bad appearance; the delirium returned, and was again successfully opposed by the same treatment.

Case V. This patient was operated upon for popliteal aneurism. He was of a plethoric and athletic constitution, and a free bleeding had been practised, to guard against accident after the operation. He appeared to be indifferent to every thing that was done, and scarcely conscious of what was passing around him. On the fifth day he was attacked with furious delirium, without fever. The symptoms were the same as those detailed in the previous cases. An anodyne enema was given with the most complete success. The ligature, however, came away from the femoral artery prematurely, and the patient expired on the fortieth day, after many attempts had been made to arrest the hemorrhage.

Case VI. In this case the patient had attempted self-destruction by cutting her throat. Delirium afterwards occurred, without fever or symptoms of inflammation. Anodynes, internally given, were effectual in relieving it.

M. Helis is not aware that any author has paid particular attention to this species of delirium. He has only found one example of it in books. Many surgeons have, indeed, spoken of the violence of some patients after operations, and of their tearing away their dressings; but none have inquired into the cause of this kind of insanity, or have endeavoured to relieve it by any other means than coercion. The danger of so violent a kind of delirium after various accidents and operations, must be evident, and it must be a source of much gratification that we possess a remedy which is almost certain in its operation. M. Helis imagines that it may generally be possible to predict the occurrence of this mental disturbance, either from the nature or duration of the operation, the character of the patient, his moral energy or physical disposition. There are some symptoms from which delirium may be almost certainly anticipated. If, a few hours, or one or two days after a fracture, an attempt to commit suicide, or any operation, the patient appears unusually gay, talks much, has a quick expression of the eyes, gives short answers, moves quickly and without any obvious motive; if he affects great courage and resolution, the surgeon should be upon his guard. The slightest excitement should be avoided. The patient should be kept in the most complete repose, away from light, noise, or unnecessary visits, or his symptoms will quickly become more decided. He will soon begin to talk unconnectedly: at one moment his language will be mild, at another violent: his loquacity will be incessant. In this state the patient is dangerous to others and to himself. In one case M. Helis mentions, a man rose in the middle of the night, and beat many of his companions with his crutch, and would probably have destroyed some of them if he had not been secured. In some instances patients have precipitated themselves from a window, or have destroyed themselves in a still more horrible manner.

The most remarkable circumstance in the midst of so much disturbance of the mental faculties, is the tranquil state of the circulation, and the absence of febrile symptoms. The patient is furious, has lost all command of himself; his face is bathed with sweat, his eyes are unusually brilliant, he cries out vociferously, and might be thought to be labouring under the most ardent phrensy; but his pulse is calm and regular, and the state of the skin removes all suspicion of inflammation. It is, in fact, a true mania, differing only from ordinary cases in its duration. M. Helis has rarely seen the attack last longer than five or six days.

The mode of subduing it is as simple as it is efficacious. It consists of a few drops of laudanum administered in a clyster. It is this remedy which M. Dupuytren constantly employs, and it is far preferable to every other. Five or six drops of laudanum given in a small clyster produce more effect than thrice the quantity taken by the stomach. This fact may be explained by the sympathy which unites the brain with the rectum. As a proof that this sympathetic connexion is not imaginary, we may cite various cases of pains in the head, delirium from constipation, the clear and active state of the mind which follows a required evacuation from the bowels, and instances of hemicrania that have resisted every other treatment, and which have yielded, as if by enchantment, to irritants placed in the rectum. But a physiological explanation may be adduced. The stomach, destined to elaborate the first element of nutrition, is endowed with a digestible power, and with secretions which alter more or less every substance which comes in contact with it; and many medicines introduced into the stomach are ineffectual, because they are mixed with the food, or their powers are weakened by the gastric juice. Hence various medicines, particularly of the vegetable class, are uncertain in their operation, or totally inefficacious with many patients. The rectum, destined to be the reservoir of the residue of digestion, absorbs, but does not digest, and it will easily be conceived that medicines which are introduced into it, provided they are not expelled, will act with more certainty than if they were administered by the stomach.*

---

The treatment recommended by M. Helis in cases of traumatic delirium, has been practised with much success in several of the London hospitals, but we know it is not yet duly appreciated by the profession at large. Two instances have occurred to us, in which anodynes had been given by the mouth without benefit. In both, the exhibition of an enema with fifteen drops of laudanum speedily quieted the turbulence and incessant loquacity of the patients; a calm sleep followed, and health was soon restored.—Editor.

